# Supplementary figures and images for: Trimester-specific phthalate exposures in pregnancy are associated with circulating metabolites in children
Source: PLoS One. 2022 Aug 30;17(8):e0272794. doi: 10.1371/journal.pone.0272794 (PMC9426875; doi:10.1371/journal.pone.0272794)

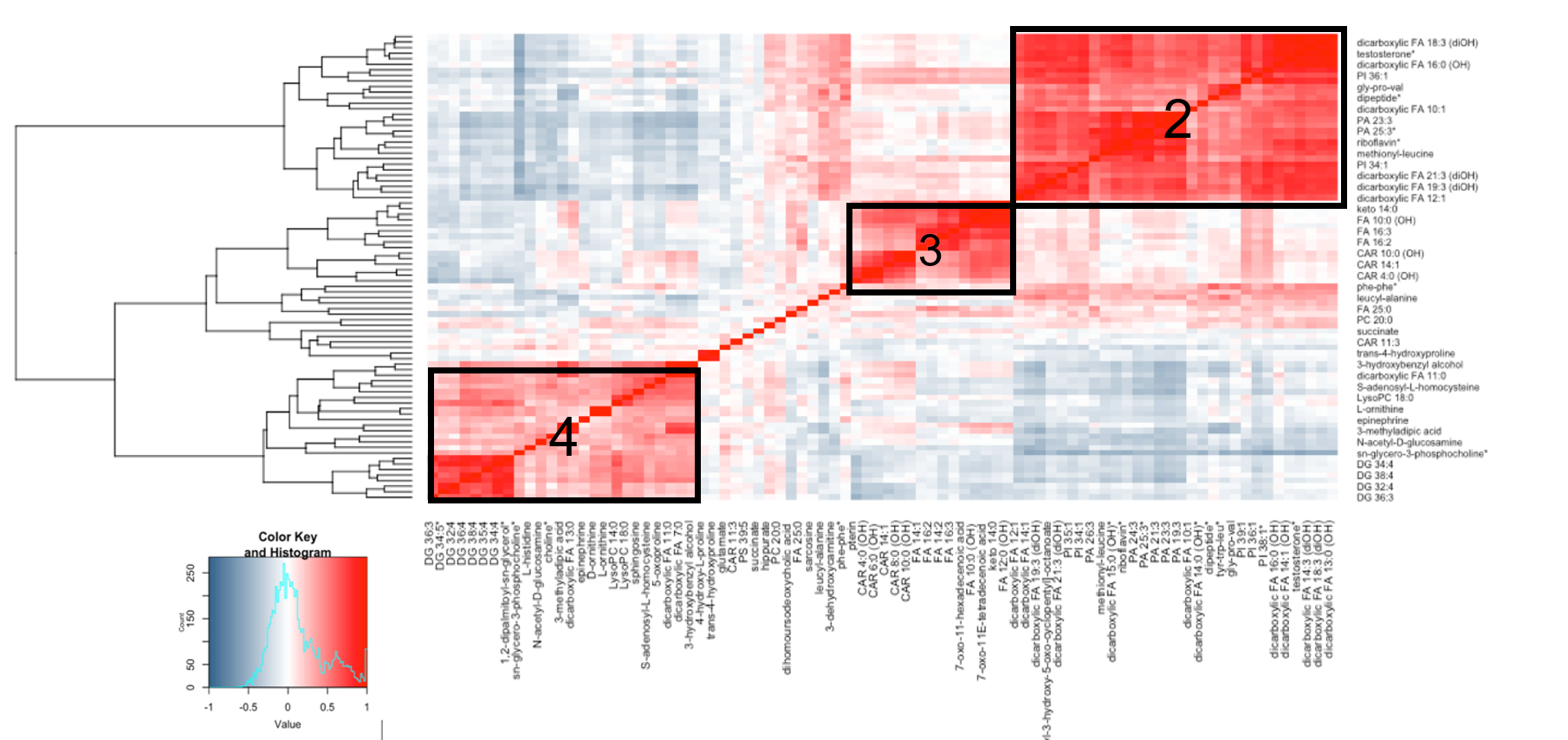

Supplement: S1 Fig — Metabolites were associated with T3 MEHHP exposure among girls at an uncorrected p-value<0.05 are included in the heatmap below; an asterisk * is next to the name of metabolites significant at q-value<0.1. The heatmap shows the Pearson correlation between these metabolites with each other, and metabolites are ordered by hierarchal clustering. (TIF) [file pone.0272794.s001.tif]

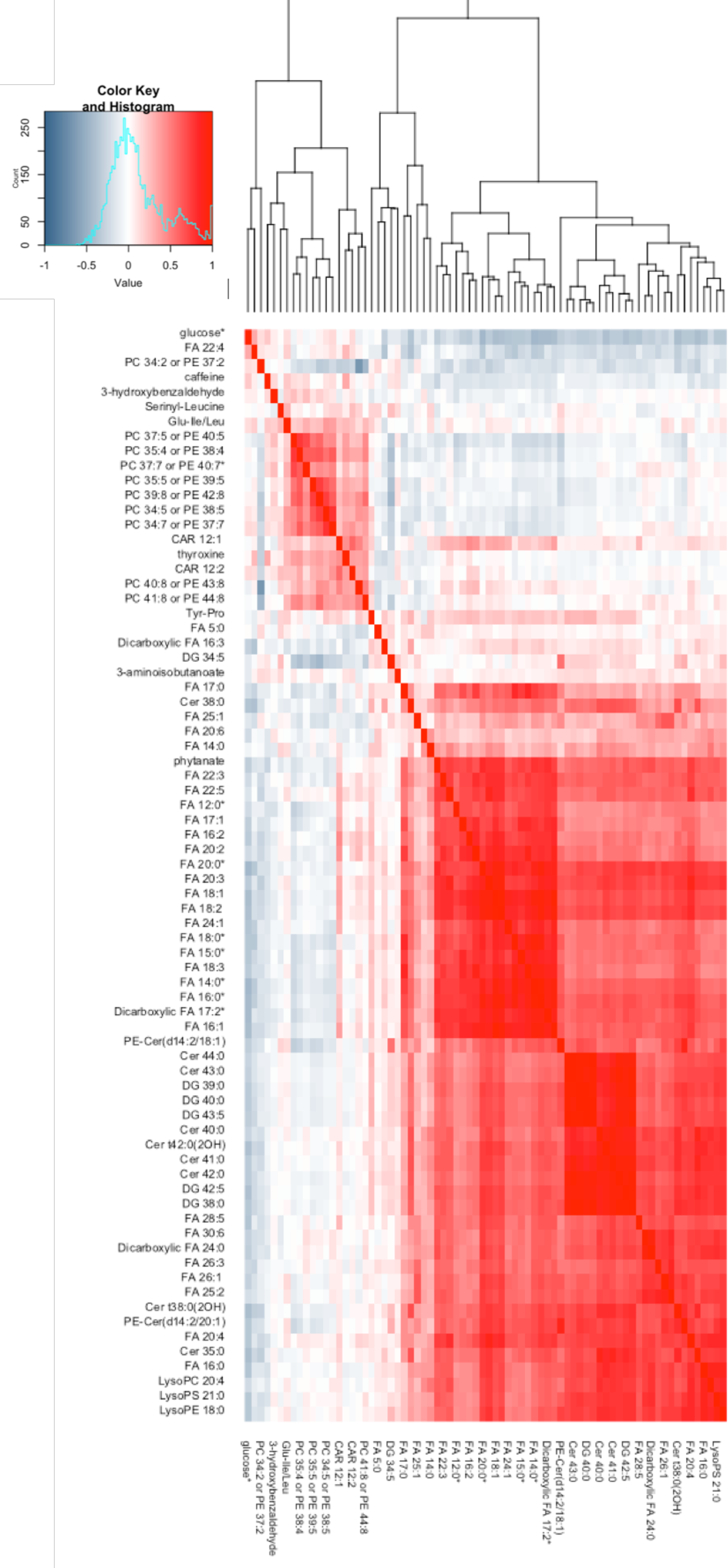

Supplement: S2 Fig — Metabolites that were associated with T3 MiBP exposure among boys at an uncorrected p-value<0.05 are included in the heatmap below; an asterisk * is next to the name of metabolites significant at q-value<0.1. The heatmap shows the Pearson correlation between these metabolites with each other, and metabolites are ordered by hierarchal clustering. (TIF) [file pone.0272794.s002.tif]
