# Supplementary material for: Trimester-specific phthalate exposures in pregnancy are associated with circulating metabolites in children
Source: PLoS One. 2022 Aug 30;17(8):e0272794. doi: 10.1371/journal.pone.0272794 (PMC9426875; doi:10.1371/journal.pone.0272794)
Supplement: S1 Table — (DOCX) [file pone.0272794.s003.docx]

**Table S1. Trimester-Specific Maternal Urinary Phthalate Concentrations Before and After Imputation**

|  | **Before Imputation** |  |  | **After imputation*** |  |
| --- | --- | --- | --- | --- | --- |
|  | **Median (ng/mL)** | **IQR (ng/mL)** | **N Imputed** | **Median (ng/mL)** | **IQR (ng/mL)** |
| ***Trimester 1:*** |  |  | 34 |  |  |
| BPA | 1.20 | (0.67, 2.3) |  | 1.20 | (0.71, 1.97) |
| MEP | 156.83 | (64.77, 410.6) |  | 167.71 | (69.24, 362.9) |
| MBP | 66.43 | (33.29, 172.7) |  | 68.87 | (37.01, 158.2) |
| MiBP | 1.20 | (0.34, 3.23) |  | 1.10 | (0.45, 2.63) |
| MBzP | 2.98 | (1.33, 6.57) |  | 3.23 | (1.49, 6.22) |
| MCPP | 1.28 | (0.77, 2.32) |  | 1.35 | (0.84, 2.25) |
| MEHP | 5.99 | (3.1, 10.56) |  | 6.01 | (3.41, 10.56) |
| MEHHP | 21.49 | (10.79, 37.93) |  | 21.40 | (12.23, 37.19) |
| MECPP | 35.53 | (24.46, 57.21) |  | 35.70 | (25.18, 55.01) |
| MEOHP | 11.09 | (5.73, 22.1) |  | 11.30 | (6.24, 20.83) |
| ∑DEHP** | 74.43 | (45.97, 128.9) |  | 74.80 | (49.06, 127.9) |
| ***Trimester 2:*** |  |  | 34 |  |  |
| BPA | 0.99 | (0.66, 1.59) |  | 0.98 | (0.66, 1.49) |
| MEP | 118.63 | (63.38, 255.0) |  | 117.41 | (67.25, 249.9) |
| MBP | 55.53 | (26.41, 111.2) |  | 56.67 | (30.64, 109.6) |
| MiBP | 0.74 | (0.25, 2.17) |  | 0.72 | (0.28, 1.91) |
| MBzP | 2.77 | (1.45, 6.18) |  | 2.74 | (1.56, 4.97) |
| MCPP | 1.16 | (0.76, 1.85) |  | 1.19 | (0.86, 1.8) |
| MEHP | 5.44 | (2.97, 9) |  | 5.43 | (3.17, 8.46) |
| MEHHP | 20.91 | (10.53, 35.93) |  | 19.95 | (10.78, 32.63) |
| MECPP | 34.13 | (22.09, 58.8) |  | 33.92 | (23.8, 55.03) |
| MEOHP | 12.19 | (6.44, 21.35) |  | 12.12 | (6.52, 20.03) |
| ∑DEHP** | 73.26 | (42.23, 125.1) |  | 72.60 | (43.77, 114.3) |
| ***Trimester 3:*** |  |  | 10 |  |  |
| BPA | 0.77 | (0.49, 1.24) |  | 0.76 | (0.49, 1.2) |
| MEP | 123.55 | (48.75, 241.5) |  | 123.03 | (53, 241.5) |
| MBP | 64.20 | (34.44, 119.0) |  | 63.75 | (34.79, 113.4) |
| MiBP | 2.05 | (1.17, 3.60) |  | 2.05 | (1.21, 3.56) |
| MBzP | 5.00 | (2.98, 8.40) |  | 4.86 | (3.1, 8.22) |
| MCPP | 1.30 | (0.74, 2.11) |  | 1.29 | (0.74, 2.05) |
| MEHP | 6.41 | (3.3, 9.88) |  | 6.42 | (3.31, 9.82) |
| MEHHP | 23.72 | (13.36, 39.64) |  | 23.76 | (13.78, 38.23) |
| MECPP | 36.72 | (22.94, 61.6) |  | 36.69 | (23.49, 60.04) |
| MEOHP | 13.34 | (8.03, 23.8) |  | 13.41 | (8.45, 22.22) |
| ∑DEHP** | 77.31 | (48.72, 134.7) |  | 77.48 | (51.6, 131.1) |

**Molar sum of MEHP, MEHHP, MEOHP, MECPP

*Imputation was done using the 5 nearest neighbors of each case with missing values, weighted by their average distance to the case, while borrowing information from the cohort, mother’s age, education, and marital status at pregnancy (IMPUTE R package).

IQR = interquartile range (25th percentile, 75th percentile)

MEP, MBP, and MiBP are low molecular weight while the rest represent high molecular weight phthalates.
