# Supplementary material for: Trimester-specific phthalate exposures in pregnancy are associated with circulating metabolites in children
Source: PLoS One. 2022 Aug 30;17(8):e0272794. doi: 10.1371/journal.pone.0272794 (PMC9426875; doi:10.1371/journal.pone.0272794)
Supplement: S2 Table — (DOCX) [file pone.0272794.s004.docx]

**Table S2. Number of Associations with q<0.1 between All Exposure Measures and 572 Metabolites among Children.**

| **Analyte** | **Trimester of Exposure Measure** | **All Children (n=234)** | **Males (n=110)** | **Females (n=124)** |
| --- | --- | --- | --- | --- |
| BPA | T1 | 0 | 0 | 0 |
|  | T2 | 0 | 0 | 0 |
|  | T3 | 0 | 0 | 0 |
| MEP | T1 | 1 | 0 | 0 |
|  | T2 | 0 | 0 | 0 |
|  | T3 | 0 | 0 | 0 |
| MBP | T1 | 1 | 0 | 0 |
|  | T2 | 0 | 1 | 0 |
|  | T3 | 1 | 0 | 0 |
| MIBP | T1 | 0 | 0 | 0 |
|  | T2 | 0 | 0 | 0 |
|  | T3 | 3 | 9 | 0 |
| MBzP | T1 | 0 | 0 | 0 |
|  | T2 | 0 | 2 | 0 |
|  | T3 | 0 | 0 | 0 |
| MCPP | T1 | 2 | 0 | 0 |
|  | T2 | 0 | 0 | 0 |
|  | T3 | 0 | 0 | 10 |
| MECPP | T1 | 0 | 0 | 0 |
|  | T2 | 0 | 0 | 0 |
|  | T3 | 0 | 0 | 15 |
| MEHHP | T1 | 0 | 0 | 0 |
|  | T2 | 0 | 0 | 0 |
|  | T3 | 0 | 0 | 13 |
| MEHP | T1 | 0 | 0 | 0 |
|  | T2 | 0 | 0 | 0 |
|  | T3 | 0 | 0 | 1 |
| MEOHP | T1 | 0 | 0 | 0 |
|  | T2 | 0 | 0 | 0 |
|  | T3 | 0 | 0 | 0 |
| ∑DEHP* | T1 | 0 | 0 | 0 |
|  | T2 | 0 | 0 | 0 |
|  | T3 | 0 | 0 | 3 |

*Molar sum of DEHP exposure biomarkers: MEHP, MEHHP, MEOHP, MECPP
