# Supplementary material for: Trimester-specific phthalate exposures in pregnancy are associated with circulating metabolites in children
Source: PLoS One. 2022 Aug 30;17(8):e0272794. doi: 10.1371/journal.pone.0272794 (PMC9426875; doi:10.1371/journal.pone.0272794)
Supplement: Attachment — Submitted filename: Response_to_Reviewers_Final.docx [file pone.0272794.s005.docx]

Response to Comments

Dear Dr. Garrett and reviewers,

Thank you for giving us valuable feedback on our manuscript. We believe this version of the manuscript is much improved. See below for point-by-point responses to each reviewer comment. Responses to journal/editorial requirements are at the end of this document. *Responses are italicized.* We have uploaded a track-changes version of the manuscript along with a clean, updated version. Line numbers listed in this response refer to the track changes version of the manuscript. Thanks for reconsidering this version of the manuscript for publication.

Reviewer #1:
The authors attempt to perturb biochemical pathways associated with exposure to phthalates during gestation, which influence the development of cardiometabolic risk. The goal is unclear, but I believe that the authors are looking to provide proof of concept that global metabolomics could identify new biomarkers in human subjects to elucidate the effects of exposure in later life, as mentioned later on in the manuscript.

Basic summary: Phthalates were measured during each trimester and correlated with the metabolic profiles, of the children, of the participants in later life (the author does a good job of justifying and identifying studies in animals that show similar findings to theirs). However, overall, it is not clear what their goal(s) are- leaving many unanswered questions.

*Thank you for pointing out that our goal was not clear. Our research team is moving towards a precision-health focus employing ‘omics’ technologies in epidemiological studies to better understand inter-individual response and to detect disease/toxicity before it is clinically apparent. There are several worthwhile ways to use these data, but we failed to highlight our primary reason for using metabolomics in this particular paper. We have adjusted the language throughout to ensure our primary reason is clear.*

*Prenatal phthalate exposures have been shown in other studies to be associated with longer-term cardiometabolic effects that manifest in childhood or later. However, how this occurs biologically is largely unknown, and we are not able to detect affected individuals (before they display an overt clinical outcome). This makes risk assessment of phthalates to optimally protect children against these unnecessary risks very challenging.*

*The main goal is now stated in the introduction: “Our overall goal is to elucidate biochemical pathways in childhood that were altered by prenatal phthalate exposures and may serve as the link between exposures and adverse cardiometabolic outcomes.”*

The paper lacks focus on the importance of the study. Pregnant women typically know that they should avoid smoking, alcohol, and raw fish, etc., but is everyone aware that they should probably avoid lipstick, perfumes, etc., probably not. However, if we were able to discover a biomarker related to the mechanism of phthalate exposure, how would we intervene given that the ‘damage’ has already been done? Or is the goal to search for biomarkers of cardiometabolic syndrome? I think the former, please clarify why such biomarkers are needed/how they will be helpful for clinical decision making/intervention.

*We are more interested in the former (identify biomarkers of phthalates’ toxicity). We believe the primary benefit of this information would be to inform risk assessment (i.e. policies to limit phthalate exposures to levels that are safe for all). It could additionally be used to identify strategies to promote the health of susceptible individuals who have already been exposed (i.e. Interventions to improve cardiometabolic health).*

Comments
1. Clarify the goal of the study- Is your goal to identify biomarkers for cardiometabolic exposure or to elucidate the mechanism of phthalate exposure to predict cardiometabolic disease? Initially I thought the former but as I read the manuscript the later seemed more fitting. What are the clinical markers of cardiometabolic risk?

*Our primary purpose in this study is to use metabolomics to understand the biological/physiological pathways that prenatal phthalate exposures impact in order to understand the cardiometabolic risk from exposure to these chemicals. We have now stated this goal in the introduction and changed the wording throughout to reflect that. At the end of the introduction, we state: “The overall goal is to elucidate altered biochemical pathways in childhood that may serve as the link between exposures and adverse cardiometabolic outcomes.* *We hypothesize that prenatal exposures will be associated with metabolite profiles relevant to metabolic risk among children*.”

2. Cardiometabolic disorders represent a cluster of interrelated risk factors, primarily hypertension, elevated fasting blood sugar, dyslipidemia, abdominal obesity, and elevated triglycerides. How will new biomarkers be useful/what aspect of the diagnostic decision making is hindered? Assuming we find biomarkers associated with risk how will they help with intervention?

*For the purposes of this manuscript, metabolomics is being used as a tool to understand toxicity of phthalates on children. The primary purpose is not to identify markers for diagnosis (though there is great potential for that in the field of metabolomics). We have removed language in the introduction that focused on clinical/diagnostic uses of metabolomics, so that the primary purpose is more evident.*

3. Did you have a control group? How do you know that the statistical differences were due to phthalate exposure during gestation, and not sex, age, diet, etc.? Admittedly challenging to control for in human studies!

*This is a challenging limitation of almost all environmental exposure studies in human populations, which largely rely on longitudinal, observational designs of necessity. Populations around the world experience a range of exposures to phthalates, yet designing an RCT to evaluate the impact of exposure vs. non-exposure would be inappropriate given the growing body of research documenting health effects. In a cohort study such as ELEMENT, individuals with very low exposures (below the limit of detection that our analytical methods can measure which are values close to 0) can however be regarded as a relevant comparison “non-exposed” group. Since chemicals often have a dose-response relationship with adverse health outcomes, we model phthalate concentrations as continuous variables to assess this relationship, expecting that relationships between phthalates and metabolites will be dose-dependent. We ran models stratified by sex, allowing for the possibility that effects could be different in boys and girls in this age group. Further, we adjusted for several potential confounders in the regression models including age, pubertal onset (yes/no), and BMI (z-score for child’s age and sex). We did not adjust for diet or current/ongoing exposures to phthalates which could have confounded the relationship, and we have added a statement about this in the limitations section (ln 392).*

4. Looks like a lot of negative ion FA’s being identified in the male (boy) cohort- have you looked at %CV variability of each given that smaller molecules do not always ionize well in negative mode?

*Thanks for this suggestion. Running metabolomics in positive and negative ionization models allows for a more complete view of the metabolome. In our analysis, all free fatty acids were identified in negative mode (45 metabolites). Fatty acid intermediates (dicarboxylic or hydroxy) were identified in both positive and negative ionization mode with more identified in the negative mode. We screened relative standard deviations (RSDs, similar to %CV) of all data from both modes prior to analysis. Features with high pooled RSDs were removed from the analysis.*

5. What is the distribution of the female (girl) cohort in terms of age? Testosterone is noted as being statistically significant but may also be due to age. The clinical lab applies different age-based reference intervals for total- and free-testosterone. Might be useful to report tanner staging results by sex in the supplemental in addition to age distribution of females (girls).

*Based on your comment, we added this information to the results text. Most of the girls were young and had not started puberty yet. The results now state:*

*“The median age of the 234 children was 9.9 years, and 53% were girls. Median age was the same for both boys and girls (9.9 years). Pubertal onset (Tanner stage >1) was documented in 39% of the children (32% of girls and 46% of boys).”*

6. How did you normalize the data and adjust for batch effects? More detail needed in the method section overall. What mass spectrometer was used- Thermo Tribrid IQX MS, for example. Liquid chromatography- mobile phase, gradient, column temperature, etc. What software was used for data analysis? Should break this section down into metabolomics and lipidomics as details will vary between the two.

*We have added more details to the methods section. Only untargeted metabolomics analysis was performed. This analysis can detect many classes of lipids; we did not perform a separate lipidomics analysis. Laboratory methods are now detailed as follows:*

*“The metabolome was assessed via an untargeted platform in fasting serum samples collected from ELEMENT children at the peri-pubertal study visit. Samples were immediately frozen following collection, and stored at -80°C until analysis. At the Michigan Regional Comprehensive Metabolomics Resource Core, liquid chromatography mass spectrometry (LC-MS) was used to detect 9,303 features in positive or negative mode (1). Briefly, metabolites were extracted using a solvent of 1:1:1 Methanol : Acetonitrile : Acetone with internal standards using 100 ml extraction solvent and 4 ml internal standard mixture. The 1290 Infinity Binary liquid chromatography system (Agilent Technologies, Inc., Santa Clara, CA) was used for chromatographic separation together with Waters Acquity UPLC HSS T3 1.8 µm 2.1 x 100 mm column in connection with a Water Acquity UPLC HSS T3 1.8 µm VanGuard pre-column. Samples were reconstituted with solvent (Methanol : Water, 2 : 98). Total run time was 34 minutes with a flow rate of 0.45 mL/min and column temperature of 55 degrees C. The 6530 Accurate-Mass Q-TOF (Agilent Technologies, Inc., Santa Clara, CA) with a dual ASJ ESI ion source was used as the mass detector. Mass spectrometry was run in positive and negative ionization modes; the positive mode was run first, and six samples had missing negative mode data due to lack of remaining sample.*

*Raw data processing identified chromatographic peaks representing metabolite features using a modified version of existing commercial software (Agilent MassHunter Qualitative Analysis). After the removal of redundant compounds or those missing in >70% of samples, 3,758 features remained (2). Metabolites were annotated by matching MS/MS fragmentation patterns, retention times, and ionization masses to metabolites within the laboratory’s compound library, allowing for the annotation of 572 annotated compounds (denoted as ‘known’) that were used for analysis hereafter. Lipids are reported with the nomenclature as X:Y, where X is the length of the carbon chain and Y is the number of double bonds.”*

*In terms of batch effect adjustment and normalization, these methods are also detailed in the methods section as follows:*

*“Prior to statistical analysis, metabolomics data were normalized, adjusted for batch effects, and missing values were imputed within each model (3). Within-batch peak intensities were adjusted for batch drift using LOESS regression; the extent of batch drift was determined by pooled samples used as quality control in each batch. Across-batch peak intensities were adjusted for drift by a multiplicative factor of the ratio between the median intensity of the batch-specific quality control samples and the median intensity of all quality control samples. Missing metabolite values were imputed using the K-nearest-neighbour algorithm (K=5) with the IMPUTE package in R from Bioconductor. Finally, each metabolite was normalized (log transformed and then standardized to mean 0 and variance 1).”*

7. What extraction was carried-out per-application, metabolomics vs lipidomics?

*Please see response to comment #6. Untargeted metabolomics was performed here.*

8. Not clear what the comparisons are. When you say ‘girls only comparison? Is this between trimesters? Clarify overall comparisons that were carried-out.

*Thanks for pointing out that our language was confusing. We have changed the wording in various places where the intent of the model was unclear. Briefly, in all models, the relationship of interest was the association between a phthalate (continuous measure, concentration measured in urine from one trimester) with a metabolite (continuous measure of relative abundance). We model these relationships in all children. We also model these relationships in boys-only and in girls-only. This stratification was decided a priori due to previous studies showing differences in the impacts of phthalate exposures on cardiometabolic outcomes by sex.*

Reviewer #2:

This manuscript reports associations between 9 maternal urinary phthalate metabolites and untargeted metabolomics profiles in serum samples collected from children aged 8-14 years. The results present a large number of phthalate associations across trimesters with serum metabolites among the entire cohort of children as well as within gender/sex. The primary weakness of the paper include:
1. the introduction could be consolidated to focus on the underlying biology of maternal phthalate exposure and pediatric outcomes. As written, the introduction is very long and provides information that is not specific to the research objective. It seems as though some of this information could be moved to the discussion.

*We have shortened the introduction (by about one-third of its original text) and kept the focus on these topics (in order): 1) introduction to phthalates, 2) evidence for prenatal phthalate exposures’ impact on cardiometabolic outcomes in children, 3) potential utility of metabolomics for evaluating chemical toxicity, 4) evidence for relationships between phthalates and metabolomics in rodent or human studies, 5) gaps in knowledge and aim of our study.*

2. methods are completely lacking information on how the metabolites were measured using mass spectrometry. Additionally, there is inadequate information on data processing software, code and techniques.

*Reviewer 1 also had this concern. See response to comment #6 – we have added details on the laboratory methods. We have also added additional details about data analysis and processing (which was performed in R using primarily packages from Bioconductor, specified in the methods section).*

3. it is unclear why the analysis was stratified by gender/sex rather than a formal interaction test.

*Stratified analyses were used rather than fitting an interaction model with sex by metabolite interactions because (1) the main interest of the sex-dependent analysis is not on the magnitude of the interaction (i.e., difference in association between male and female), but the association conditional on sex; (2) the ease of adjusting for multiple comparisons in the stratified models, which can be done independently for each sex, compared to in the interaction model, which would first require a transformation of the main effect terms and the interaction terms; and (3) stratification by sex allows us to examine interactions between all covariates and sex, which avoids the subjectivity in the choice of specific interaction terms to be included in the model. Hence stratified analyses were chosen over an interaction model.*

4. The discussion suffers from the same issue as the introduction. It is rambling and hard to discern what is important. I would recommend consolidating the discussion into discreet paragraphs related to: overall cohort results, sex specific results, rationale biological explanation that links underlying biology to the results.

*We have tried to streamline the discussion to stay more closely to the goals of this paper. The discussion now follows this outline: 1) overall summary of results, 2) overall cohort results, 3) girls-only results, 4) boys-only results, 5) biological rationale, 6) study limitations, 7) conclusion. Within the discussion of the results, we focus on supporting evidence from other studies that these particular metabolites may be relevant to phthalate toxicity/adverse outcomes and aimed to exclude extraneous details.*

5. results/tables would benefit from including standardized metabolite ID's (HMDB, chemspider) that will help with replicating these results in future studies.

*The challenge with untargeted metabolomics is that many of the metabolites cannot be properly mapped to a specific HMDB. For instance, untargeted metabolomics cannot determine the fatty acid tail lengths and double bond location for DG 34:5 (e.g., DG 18:2/16:3 or DG 18:3/16:2), therefore, HMDB numbers are very challenging to accurately define for all features. In response, we did include “sub pathway” labeling in the tables. We acknowledge that targeted validation should be done for top biomarkers of interest. We have also mentioned this in the limitations (ln 387-390).*

Response to Journal Requirements:

1. Please ensure that your manuscript meets PLOS ONE's style requirements, including those for file naming. The PLOS ONE style templates can be found at
   <https://journals.plos.org/plosone/s/file?id=wjVg/PLOSOne_formatting_sample_main_body.pdf> and 
   <https://journals.plos.org/plosone/s/file?id=ba62/PLOSOne_formatting_sample_title_authors_affiliations.pdf>

*We checked the style guidelines and have updated the style of our text and citation format to fit the journal’s requirements.*

2. We note that the grant information you provided in the ‘Funding Information’ and ‘Financial Disclosure’ sections do not match.

When you resubmit, please ensure that you provide the correct grant numbers for the awards you received for your study in the ‘Funding Information’ section.

*We updated the ‘Funding Information’ sections to ensure that it is correct.*

3. Thank you for stating in your Funding Statement: "This study was funded by grants from the U.S. Environmental Protection Agency (US EPA, <https://www.epa.gov/>), grant numbers RD834800 (KEP) and RD83543601 (KEP), and from the National Institute for Environmental Health Sciences (NIEHS, <https://www.niehs.nih.gov/>) P20 ES018171 (KEP), P01 ES02284401 (KEP), R01 ES007821 (KEP), R01 ES014930 (MMTR), R01 ES013744 (MMTR), and P30 ES017885 (KEP, JMG). This study was also supported and partially funded by the National Institute of Public Health/Ministry of Health of Mexico (<https://www.insp.mx/insp-overview.html>; MMTR). The contents of this publication are solely the responsibility of the grantee and do not necessarily represent the official views of the funding agency. The funders had no role in study design, data collection and analysis, decision to publish, or preparation of the manuscript."

Please provide an amended statement that declares *all* the funding or sources of support (whether external or internal to your organization) received during this study, as detailed online in our guide for authors at <http://journals.plos.org/plosone/s/submit-now>.  Please also include the statement “There was no additional external funding received for this study.” in your updated Funding Statement. 
Please include your amended Funding Statement within your cover letter. We will change the online submission form on your behalf.

*The funding information section has been double checked and it is now correct. We have also added the corrected paragraph to our cover letter to ensure the right information will be displayed when the manuscript is published.*

4. We note that you have provided funding information that is not currently declared in your Funding Statement. However, funding information should not appear in the Acknowledgments section or other areas of your manuscript. We will only publish funding information present in the Funding Statement section of the online submission form. 
Please remove any funding-related text from the manuscript and let us know how you would like to update your Funding Statement. Currently, your Funding Statement reads as follows: "This study was funded by grants from the U.S. Environmental Protection Agency (US EPA, <https://www.epa.gov/>), grant numbers RD834800 (KEP) and RD83543601 (KEP), and from the National Institute for Environmental Health Sciences (NIEHS, <https://www.niehs.nih.gov/>) P20 ES018171 (KEP), P01 ES02284401 (KEP), R01 ES007821 (KEP), R01 ES014930 (MMTR), R01 ES013744 (MMTR), and P30 ES017885 (KEP, JMG). This study was also supported and partially funded by the National Institute of Public Health/Ministry of Health of Mexico (<https://www.insp.mx/insp-overview.html>; MMTR). The contents of this publication are solely the responsibility of the grantee and do not necessarily represent the official views of the funding agency. The funders had no role in study design, data collection and analysis, decision to publish, or preparation of the manuscript. "

*We have removed funding information from the Acknowledgements section, where it doesn’t belong. Please see cover letter and responses to comments #2 and 3 above.*

5. We note that you have indicated that data from this study are available upon request. PLOS only allows data to be available upon request if there are legal or ethical restrictions on sharing data publicly. For more information on unacceptable data access restrictions, please see <http://journals.plos.org/plosone/s/data-availability#loc-unacceptable-data-access-restrictions>.

In your revised cover letter, please address the following prompts:

a) If there are ethical or legal restrictions on sharing a de-identified data set, please explain them in detail (e.g., data contain potentially sensitive information, data are owned by a third-party organization, etc.) and who has imposed them (e.g., an ethics committee). Please also provide contact information for a data access committee, ethics committee, or other institutional body to which data requests may be sent.

b) If there are no restrictions, please upload the minimal anonymized data set necessary to replicate your study findings as either Supporting Information files or to a stable, public repository and provide us with the relevant URLs, DOIs, or accession numbers. For a list of acceptable repositories, please see <http://journals.plos.org/plosone/s/data-availability#loc-recommended-repositories>.

We will update your Data Availability statement on your behalf to reflect the information you provide.

*We have discussed data availability in the cover letter and here. Note that data will be available in our institutional repository – the University of Michigan Library’s Deep Blue Data. All data (metabolomics, exposure, covariates) needed to replicate this analysis described in this paper was submitted to the repository. However, we are still waiting on receiving the accession numbers from the repository. Once we get those, we will submit them to the journal immediately (prior to publication of the article). At the time of publication, the repository will open up access to our dataset and it will be available to any interested person. When we have the accession numbers (listed as XXX right now), our data availability statement would be:*

*All data files are available from the University of Michigan Library’s Deep Blue Data repository (accession numbers: XXX ).*

6. We note that you have included the phrase “data not shown” in your manuscript. Unfortunately, this does not meet our data sharing requirements. PLOS does not permit references to inaccessible data. We require that authors provide all relevant data within the paper, Supporting Information files, or in an acceptable, public repository. Please add a citation to support this phrase or upload the data that corresponds with these findings to a stable repository (such as Figshare or Dryad) and provide and URLs, DOIs, or accession numbers that may be used to access these data. Or, if the data are not a core part of the research being presented in your study, we ask that you remove the phrase that refers to these data.

*The ‘data not shown’, where it was written, was not necessary. We have removed it. We have made data publicly available as requested (see response to comment #5).*

7. Please upload a new copy of Figures S1 and S2 as the detail is not clear. Please follow the link for more information: <https://blogs.plos.org/plos/2019/06/looking-good-tips-for-creating-your-plos-figures-graphics/>" <https://blogs.plos.org/plos/2019/06/looking-good-tips-for-creating-your-plos-figures-graphics/>

*S1 and S2 Figures have been updated according to journal specifications, using the PACE software.*

References

1.	Evans CR, Karnovsky A, Kovach MA, Standiford TJ, Burant CF, Stringer KA. Untargeted LC-MS Metabolomics of Bronchoalveolar Lavage Fluid Differentiates Acute Respiratory Distress Syndrome from Health. Journal of proteome research. 2014;13(2):640-9.

2.	Kachman M, Habra H, Duren W, Wigginton J, Sajjakulnukit P, Michailidis G, et al. Deep annotation of untargeted LC-MS metabolomics data with Binner. Bioinformatics. 2020;36(6):1801-6.

3.	LaBarre JL, Peterson KE, Kachman MT, Perng W, Tang L, Hao W, et al. Mitochondrial Nutrient Utilization Underlying the Association Between Metabolites and Insulin Resistance in Adolescents. The Journal of Clinical Endocrinology & Metabolism. 2020;105(7):dgaa260.
